# The 2023 patient profile and clinical management characteristics at a South African chiropractic training clinic: a descriptive cross-sectional study

**DOI:** 10.1186/s12998-026-00634-2

**Published:** 2026-03-25

**Authors:** Bevan Leedham, Fatima Ismail, Christopher Yelverton

**Affiliations:** Department of Chiropractic, Faculty of Health Sciences, University of Johannesburg, John Orr Building, 7th Floor, 55 Beit Street, Doornfontein, Johannesburg, 2028 South Africa

**Keywords:** Chiropractic, Education, Chiropractic, Teaching clinics, Preceptorship, Clinical competence, Demography, Retrospective studies, Cross-sectional studies

## Abstract

**Background:**

Clinical training within chiropractic education relies on exposure to diverse case mix to develop clinical competence. South African institution-based chiropractic clinics are essential learning environments and providers of affordable musculoskeletal healthcare. Periodic evaluation of patient profiles tracks evolving populations and healthcare utilisation, informing longitudinal comparisons. The purpose was therefore to describe the demographic and clinical characteristics of new patients presenting to the University of Johannesburg Chiropractic Clinic (UJCC) in 2023.

**Methods:**

A retrospective, descriptive, cross-sectional review screened all new patient files created between January 1, and June 30, 2023. Routinely documented information from initial consultation to extraction (May–July 2025) was manually captured from eligible files using a structured form, including age, sex, complaint regions, number of visits, and modalities utilised over complaint stages (primary/secondary/tertiary). Descriptive statistics (frequencies, percentages, means, standard deviations) were generated using IBM SPSS v30, with non-parametric tests applied where appropriate.

**Results:**

Of 1435 files screened, 880 were included [54% female; mean age 40.9(± 17.51) years]. Secondary and tertiary complaints were reported in 39.1% and 14.8% of patients. When aggregated across all complaint stages lumbar (41.5%) and cervical (32.8%) regions predominated. Treatment episodes were brief [primary: mean 2.31(± 2.06) sessions, 72% ≤2 visits; secondary: mean 2.88(± 2.98), 62.2% ≤2 visits; overall: mean 4.24(± 4.98), 49.6% ≤2 visits]. Management was multimodal and manual-therapy-led: manipulation/mobilisation (primary: 94.8%; secondary 98.5%), massage (68.1%; 65.1%), and myofascial dry needling (40.1%; 59.9%) used in most cases; shockwave was utilised for 15.8% of primary and 23.3% of secondary cases, while laser therapy (0%; 0.2%) was rare. Sex- and age-related patterns of regional examinations were generally consistent. Age showed a weak positive correlation with the number of secondary-complaint sessions (ρ = 0.151, *p* = 0.005, n = 344).

**Conclusion:**

UJCC’s 2023 case mix was predominantly adult, spine-dominant, featuring brief treatment courses consisting of multimodal, manual-therapy-led care with selective use of adjunctive soft-tissue and electrophysical modalities. This study provides an updated baseline for curriculum review, resource planning, and longitudinal benchmarking with other training clinics and private practices.

## Background

In the context of chiropractic education, clinical training forms the bridge between theoretical knowledge and practical experience, where students have opportunities to treat patients in a supervised clinical environment [1]. The quality of this experience depends on the diversity and volume of patient presentations encountered, referred to as the clinical case mix [2], which is indispensable for cultivating clinical competence, diagnostic acumen, and confidence in managing a wide range of musculoskeletal and associated health conditions [3]. Heterogeneous clinical profiles are therefore essential to ensure that chiropractic graduates achieve the competence and adaptability required for independent practice [4, 5].

Institution-based chiropractic clinics occupy a pivotal role in both professional education and community healthcare delivery. Within these educational clinic settings, students provide affordable evidence-informed healthcare to the public while functioning as supervised learning environments [4]. In South Africa, where chiropractic services remain largely excluded from the public healthcare sector [6, 7], the affordable chiropractic care offered to the public by institution-based training clinics serves as a vital point of access to conservative musculoskeletal healthcare [4]. The University of Johannesburg (UJ) is one of two tertiary institutions in Africa that offer a chiropractic programme [8]. At the University of Johannesburg Chiropractic Clinic, students enrolled in the Master of Health Sciences (MHSc) in Chiropractic gain practical experience and clinical exposure to patients of varied socioeconomic backgrounds, preparing them for private and public healthcare settings [4]. Consequently, the characteristics of patients presenting for chiropractic care at these facilities have direct implications for the development of well-rounded chiropractic practitioners [2, 9].

A previous retrospective study conducted at the University of Johannesburg Chiropractic Clinic (UJCC) characterised the demographic profile, regional distribution of complaints, and treatment patterns of new patients attending the clinic in 2016 [4]. That study reported a predominantly younger patient population and a case mix largely dominated by lumbar and cervical presentations and brief treatment episodes, while patient sex was not recorded. These findings established an important baseline profile of the clinic’s case mix and informed understanding of clinical exposure within this educational setting at that time. Other studies conducted at similar chiropractic training clinics have consistently identified patient populations with a slight female predominance, specifically adults in their third to fifth decades, despite large cohorts representative of student populations, with a prevalence of lumbar and cervical regional complaints [4, 5, 9–12].

However, population demographics, clinic utilisation patterns, and patient management strategies evolve over time, potentially altering the profiles of patients who seek chiropractic care at educational institution-based clinics. Regularly updating these data is therefore necessary to identify longitudinal trends and evaluate whether the clinical exposure that students receive offers a diverse range of patient populations, complaint regions encountered, and treatment modalities utilised [4].

Furthermore, few studies at educational institution-based chiropractic clinics have investigated an extensive range of treatment modalities, their usage frequencies, and treatment session frequencies per complaint and total, beyond the initial presenting complaint. These parameters not only reflect patient management practices and continuity of care but also provide insight into the optimisation of resource allocation, curriculum alignment, and contribution to the evidence base on modality usage frequencies in educational clinic settings.

Accordingly, the present study aimed to describe the demographic and clinical characteristics of new patients who presented to the University of Johannesburg Chiropractic Clinic in the first six months of 2023. Specifically, the study examined patient age and sex distributions, the regions of primary, secondary, and tertiary complaints, the number of treatment sessions associated with each complaint, and the treatment modalities utilised, along with their frequencies across these complaints. By providing this expanded and updated profile of this patient population and comparing it with earlier findings, this study contributes to creating a comprehensive body of research characterising South African chiropractic training clinic populations and the chiropractic care they provide.

## Methods

### Study design and setting

This study adopted a retrospective, descriptive design, reviewing all new patient files created between January 1, 2023, and June 30, 2023, at the University of Johannesburg Chiropractic Clinic. Using clinical data routinely captured in patient files during chiropractic consultations, data were extracted for the review period, from the initial consultation in the first half of 2023 to the point of data extraction, which occurred between May 23, 2025, and July 31, 2025. At the time of the study, UJCC utilised exclusively paper-based patient records. Files consisted of physical folders containing handwritten clinical notes, structured regional examination forms, treatment records, and signed informed consent documentation. Electronic health record systems were not in use during this study. These patient files were located and stored within the clinic’s secure filing room, located on the Doornfontein campus in Johannesburg, South Africa.

### Population and sample size

All new patient files created between January 1, 2023, and June 30, 2023, formed the study population. Files were screened against eligibility criteria. Eligibility required that the file be present in file storage during data extraction and corresponded with chiropractic care, with the initial consultation date falling within the study period, the first six months of 2023. Notably, the signed patient informed consent document and treatment plan (SOAP) needed to be present and completed for analysis inclusion.

### Data collection and analysis

Patient files, when created, are issued with sequential numerical identifiers and stored accordingly. Starting with the first eligible file created in 2023, each file was systematically screened for eligibility, and the documents contained within were reviewed to capture relevant information onto a structured data collection form. The structured data collection form was developed to standardise extraction from physical patient records. The form captured variables directly aligned with this study and prior studies, thereby supporting longitudinal comparability. The data collection form captured patient age, sex, primary, secondary, and tertiary regions of complaint, the number of treatment sessions per primary and secondary complaint, treatment modalities utilised, and their frequencies per primary and secondary complaint stages. Additionally, the total number of treatment sessions each patient received up to the point of data extraction, ending in July 2025, was recorded. Data fields within included files were completed for the core descriptive variables reported (age, sex, complaint region). If a specific modality field was left blank, it was recorded as ‘not used’. All variables were defined as recorded in the standard clinic documentation forms. Complaint regions were recorded by the treating intern based on patient history and physical examination. Treatment modalities were recorded as administered per session.

The primary complaint was defined as the patient’s presenting complaint at the initial consultation. For each complaint stage (primary, and where applicable secondary/tertiary), the regional examinations documented in the file were recorded. Therapeutic modalities were extracted from treatment notes at the session level: each modality was recorded as present/used if it occurred during that visit and tallied for the duration of the complaint until a new complaint was encountered. Repeated use of the same modality within a single session (applied more than once or to multiple regions) was recorded only once for that session. If a new complaint arose later, it was captured as a secondary (or tertiary) complaint stage, with the same regional examination and session-level modality recording approach applied to its subsequent visits. Treatment sessions (visits) were defined as documented encounters in the SOAP notes and other supporting attendance documents. Session counts were summarised by complaint stage (primary, secondary, tertiary) because complaints were tracked as distinct clinical episodes over time indicated by the completion of new complaint and regional examination documentation.

Data analysis was conducted in collaboration with Statistical Consultation Services (STATKON) using IBM SPSS software version 30 (IBM Corp., Armonk, NY, USA). Descriptive statistics, including frequencies, percentages, means, medians, standard deviations, and ranges, were generated to summarise demographic and clinical variables. Exploratory cross-tabulations were used to examine relationships between demographic and clinical variables, such as age group, sex and complaint region. However, inferential tests could not be applied where data involved non-mutually exclusive multiple-response variables. The normality of continuous variables was assessed using the Kolmogorov-Smirnov test with Lilliefors’ correction. Where normality assumptions were violated, non-parametric analyses, including Spearman’s rank-order correlation, were performed. Statistical significance was set at *p* < 0.05 for all tests.

### Reliability and validity

Data were extracted by a single researcher for consistency using a structured collection form developed for this research. The form was piloted using ten files and then refined: coded reasons for exclusion were added, the layout was revised to improve data capture efficiency, and additional treatment modalities were incorporated into the relevant sections. Piloted files were not included in the final analysis. Content validity was established through alignment of variables with the study objectives and prior published chiropractic teaching-clinic studies. The form was reviewed by research supervisors to ensure completeness before implementation. To enhance intra-examiner consistency, approximately 10% of files were randomly rechecked by the researcher, with no variation found between entries. Formal reliability coefficients were not calculated. Formal inter-examiner reliability statistics were not applicable, as extraction was performed by a single researcher. The extracted data were aggregated into a Microsoft Excel spreadsheet (Microsoft Corporation, Redmond, WA, USA) and verified for accuracy and completeness before statistical analysis. These measures collectively ensured accuracy, reliability and validity.

### Ethical considerations

Institutional approval (REC-3369-2025) was obtained prior to data collection, and permission to use patient files for research was granted by the dean and respective institutional authorities. No patient contact occurred due to the retrospective nature of this study, which collected only de-identified, routinely recorded clinical data. All data extraction occurred in the clinic’s secure file storage room during operational hours; no records were removed from the premises.

Prior to data extraction, files were assigned a unique study code and screened to ensure that the informed consent document was completed by the patient, indicating that information from records may be utilised for research purposes with anonymity retained. All de-identified data were securely stored on an encrypted, password-protected device using structured data collection forms on Microsoft Word (Microsoft Corporation, Redmond, WA, USA) and reported in aggregate with a Microsoft Excel spreadsheet (Microsoft Corporation, Redmond, WA, USA), accessible only to the researcher and statistician for subsequent analysis. Well-being, privacy, and confidentiality were maintained throughout; no conflicts of interest were reported.

## Results

**Fig. 1:**
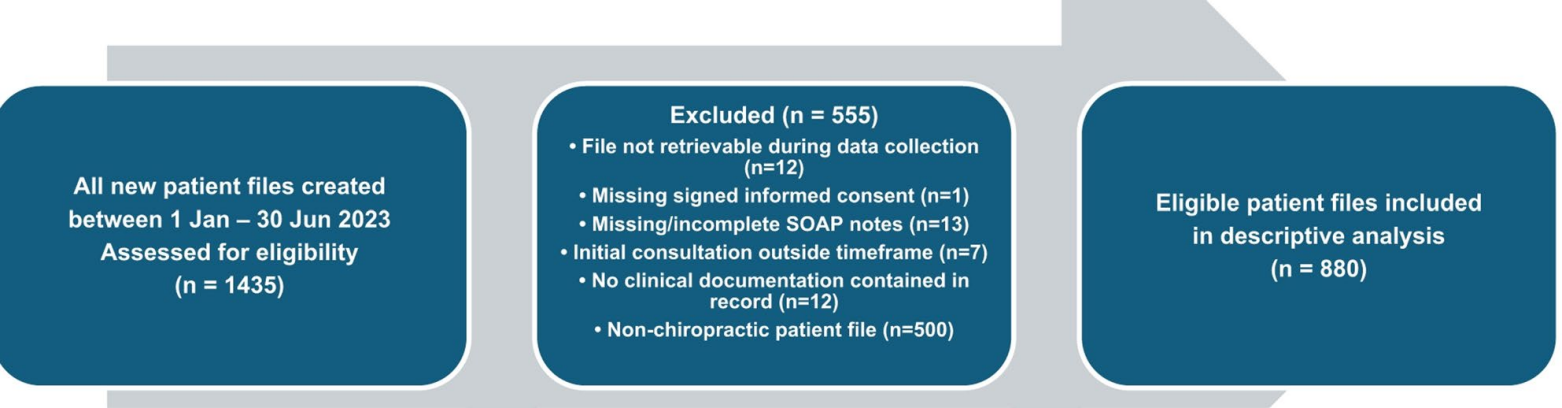
Flow diagram illustrating the screening and inclusion process for patient records

A total of 1435 new patient files were reviewed for eligibility, of which 880 files were included in analyses (Fig. 1). The results are presented in six sections: Demographic data, complaint regions, treatment frequencies, therapeutic modalities, crosstabulations, and normality and correlation.

### Demographic data

The sex distribution indicated a slight predominance of female patients at 54%. The median age of patients was 39 years (IQR:27–55) with a range of 9 to 87 years and a modal age of 23 years (3.6%; n = 32). Age was recorded as continuous raw data and subsequently grouped into three categories (< 30; 30–49; ≥50 years) following statistical consultation for efficient data presentation. When categorised, the largest proportion of patients were aged 30–49 years (35%, n = 308), as indicated in Table 1. Patients aged 18–23 years represented 19.5% (n = 172) of the sample, while those aged 65–87 years represented 12.3% (n = 108), and those aged 9–17 years represented 2.3% (n = 20).

**Table 1:** Frequency of demographic variables (n = 880)

Demographic characteristic	n (%)
Sex	
Female	475 (54%)
Male	405 (46%)
Age group (years)	
< 30	297 (33.7%)
30–49	308 (35%)
≥ 50	275 (31.3%)

Note: Data presented as n (%). Age groups: <30, 30–49, ≥50 years

### Complaint regions

From the sample, all (n = 880) had primary complaints, after which 39% (n = 344) reported secondary complaints, and 14.8% (n = 130) later reported tertiary complaints. Across the 1354 primary, secondary, and tertiary complaints within this study, a total of 1825 regional examinations were recorded, as shown in Table 2, indicating that on average more than one (mean = 1.35) regional examination documents were recorded per complaint episode.

**Fig. 2:**
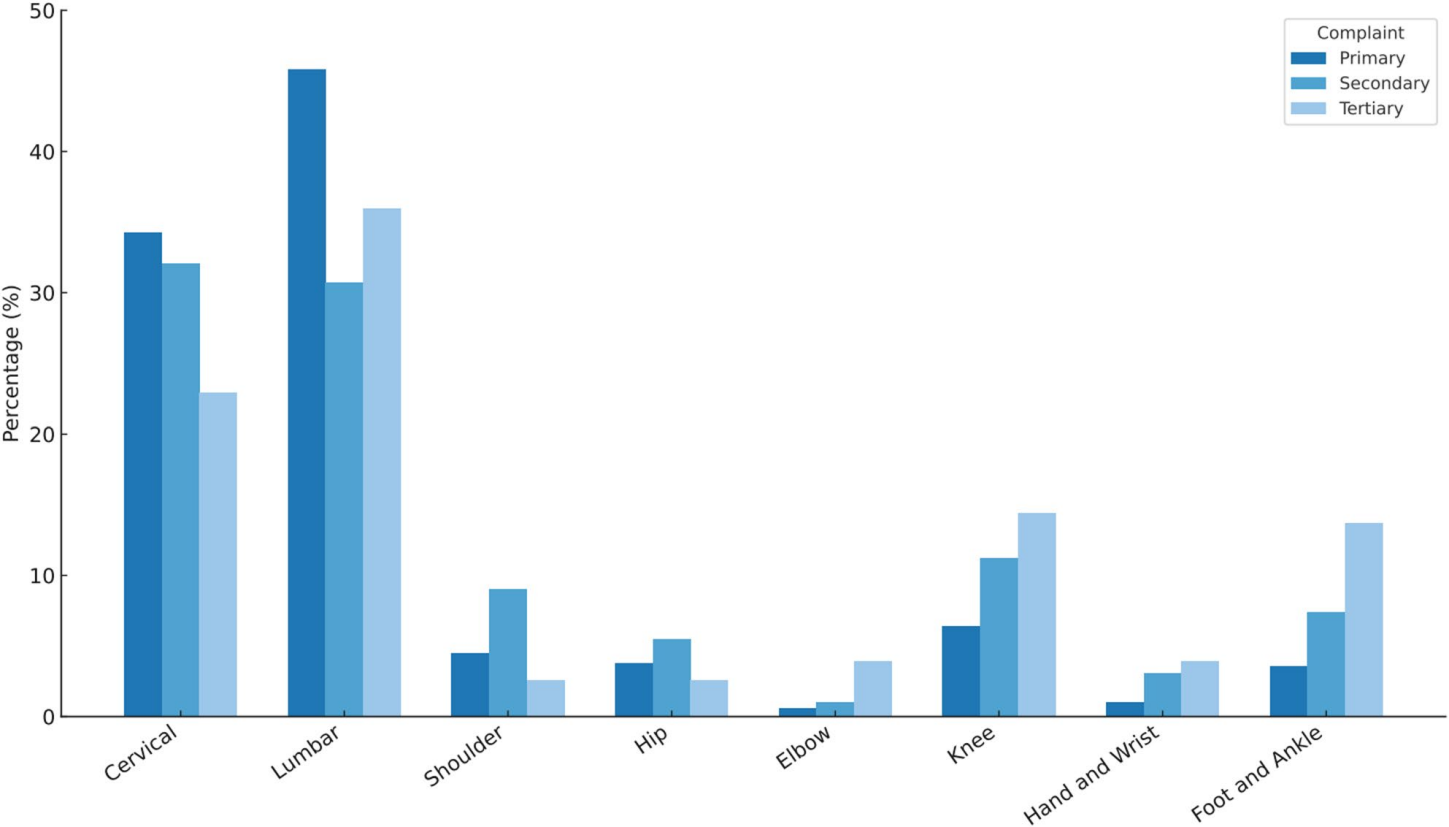
Distribution of regional complaints across primary, secondary, and tertiary presentations

When regional examinations were considered in aggregate (across all complaint stages), the lumbar region accounted for the largest proportion (41.5%; n = 758), followed by the cervical region (32.8%; n = 599), together comprising 74.3% of recorded regional examinations. Extremity regional examinations constituted the remainder (25.7%; n = 468), most commonly the knee (8.2%; n = 149), shoulder (5.4%; n = 98), and foot/ankle (5.3%; n = 97), followed by the hip (4.1%; n = 75). The least frequently recorded regions examined were the hand/wrist (1.8%; n = 32) and elbow (0.9%; n = 17). Figure 2 illustrates the proportional distribution of regional examinations performed across complaint stages for the full dataset.

**Table 2:** Regional examinations for complaints n (%)

Region	Primary	Secondary	Tertiary	Overall
Cervical	429 (34.3)	135 (32.1)	35 (22.9)	599 (32.8)
Lumbar	574 (45.8)	129 (30.7)	55 (36.0)	758 (41.5)
Shoulder	56 (4.5)	38 (9.0)	4 (2.6)	98 (5.4)
Hip	48 (3.8)	23 (5.5)	4 (2.6)	75 (4.1)
Elbow	7 (0.6)	4 (1.0)	6 (3.9)	17 (0.9)
Knee	80 (6.4)	47 (11.2)	22 (14.4)	149 (8.2)
Hand and Wrist	13 (1.0)	13 (3.1)	6 (3.9)	32 (1.8)
Foot and Ankle	45 (3.6)	31 (7.4)	21 (13.7)	97 (5.3)
Total	1252	420	153	1825

Note: Values presented as n (%). Column percentages. Regional examinations exceed complaints due to multiregional assessments

### Treatment frequencies

A total of 3731 treatment sessions were recorded for this sample, between January 1, 2023, and July 31, 2025, of which there were 2035 (54.5%) treatment sessions for primary complaints, 990 (26.5%) for secondary complaints, and the remaining 706 (18.9%) for subsequent complaints. The number of treatment sessions varied substantially among patients, as shown in Table 3, ranging from 1 to 45 total visits. Specifically, 63.4% (n = 558) of the sample attended three or fewer sessions, while a small population (8.9%; n = 78) attended ten or more sessions overall.

**Fig. 3:**
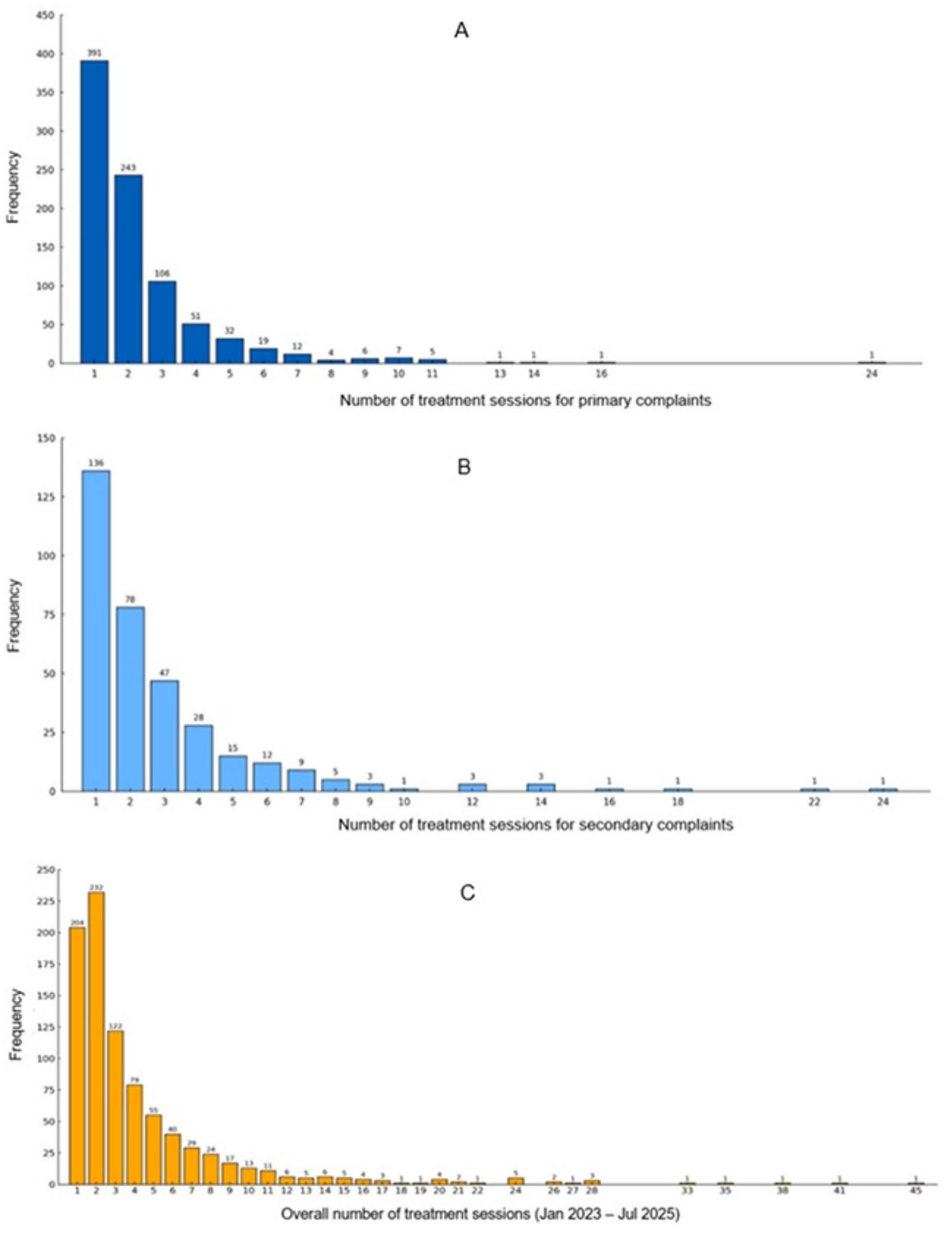
Distribution of treatment frequencies for the primary complaint (**A**), secondary complaint (**B**), and overall between January 2023, and July 2025 (**C**)

For primary complaints (n = 880), most patients received one (44.4%; n = 391) or two (27.6%; n = 243) treatment sessions, with progressively fewer receiving higher treatment frequencies. The cumulative percentage indicated that 89.9% (n = 791) of patients had four or fewer sessions during primary-complaint care. Among secondary complaints (n = 344), a similar pattern emerged: one treatment session was recorded for 39.5% (n = 136), two sessions for 22.7% (n = 78), and three sessions for 13.7% (n = 47) of the secondary-complaint cases. The cumulative proportion of patients who received four or fewer sessions for secondary-complaint care was 84.0% (n = 289). Figure 3 illustrates the distribution of treatment sessions received for the primary complaint, secondary complaint, and the overall number of treatment sessions this sample received until data extraction, which ended in July 2025.

**Table 3:** Treatment session frequencies n (%)

Number of treatment sessions	Primary complaint n (%)	Secondary complaint n (%)	Total treatment sessions n (%)
1	391 (44.4)	136 (39.5)	204 (23.2)
2	243 (27.6)	78 (22.7)	232 (26.4)
3	106 (12.0)	47 (13.7)	122 (13.9)
4	51 (5.8)	28 (8.1)	79 (9.0)
5–9	73 (8.3)	44 (12.8)	165 (18.8)
10+	16 (1.8)	11 (3.2)	78 (8.9)
Total (n)	880	344	880
Mean (SD)	2.31 (± 2.06)	2.88 (± 2.98)	4.24 (± 4.98)
Median (Mode)	2 (1)	2 (1)	3 (2)
Range (min-max)	1–24	1–24	1–45

Note: Data presented as n (%). Column percentages. Treatment sessions defined as documented encounters per complaint stage

#### **Therapeutic modalities**

In aggregate, 5469 modality implementations were recorded, comprising 3748 for primary complaints and 1721 for secondary complaints. Among the modalities used for primary complaints, shown in Table 4, chiropractic manipulation/mobilisation was utilised in 94.8% (n = 834) of primary complaint cases, followed by massage (68.1%), and myofascial dry needling (40.1%). Electrotherapies, such as interferential current (30.9%), ultrasound (26.1%), and shockwave (15.8%), were most commonly used in the middle third of modalities employed in primary complaint cases; laser therapy was never utilised. An average of ≈ 4.26 modalities were utilised across the period of each primary complaint case. For the 344 secondary complaint cases, shown in Table 5, chiropractic manipulation/mobilisation was utilised in 98.5% of the secondary complaint cases, followed by massage (65.1%), and myofascial dry needling (59.9%). Laser therapy was only used twice. An average of ≈ 5 modalities was utilised over the course of each secondary complaint case.

**Table 4:** Treatment modalities utilised for primary complaints (n = 880)

Modality	n (%)	Mean (SD)	Min-Max	% Use for primary complaint cases
Manipulation/mobilisation	834 (22.3)	2.29 (2.08)	1–24	94.8%
Shockwave	139 (3.7)	2.20 (2.09)	1–14	15.8%
Dry needling	353 (9.4)	1.94 (1.94)	1–22	40.1%
Ultrasound	230 (6.1)	1.95 (1.49)	1–10	26.1%
IFC	272 (7.3)	1.87 (1.36)	1–9	30.9%
Massage	599 (16.0)	1.73 (1.25)	1–10	68.1%
Distraction	279 (7.4)	1.79 (1.54)	1–13	31.7%
Active/passive release	343 (9.2)	1.69 (1.24)	1–10	39.0%
Ischemic compression	272 (7.3)	1.53 (1.08)	1–11	30.9%
IASTM	98 (2.6)	1.76 (1.18)	1–7	11.1%
PNF	57 (1.5)	1.56(1.15)	1–8	6.5%
Taping/strapping	80 (2.1)	1.35 (0.80)	1–5	9.1%
Thermo-/cryotherapy	131 (3.5)	1.56 (1.30)	1–13	14.9%
Cross friction	61 (1.6)	1.44 (0.67)	1–4	6.9%
Laser	0 (NA)	NA	NA	NA
Total	3748 (100%)	–	–	425.9%

Note: % Use = percentage of patients receiving modality ≥1 time during primary complaint care. IFC, interferential current; IASTM, instrument-assisted soft tissue mobilisation; PNF, proprioceptive neuromuscular facilitation

**Table 5:** Treatment modalities utilised for secondary complaints (n = 344)

Modality	n (%)	Mean (SD)	Min–Max	% Use for secondary complaint cases
Manipulation/mobilisation	339 (19.7)	2.86 (2.97)	1–24	98.5%
Shockwave	80 (4.6)	2.36 (2.14)	1–12	23.3%
Dry needling	206 (12.0)	2.44 (2.36)	1–20	59.9%
Ultrasound	136 (7.9)	2.14 (1.87)	1–15	39.5%
IFC	101 (5.9)	1.99 (1.73)	1–12	29.4%
Massage	224 (13.0)	2.26 (2.31)	1–21	65.1%
Distraction	122 (7.1)	2.33 (2.54)	1–18	35.5%
Active/passive release	140 (8.1)	2.29 (2.22)	1–16	40.7%
Ischemic compression	138 (8.0)	1.77 (1.43)	1–12	40.1%
IASTM	74 (4.3)	2.09 (2.17)	1–12	21.5%
PNF	22 (1.3)	1.27 (0.63)	1–3	6.4%
Taping/strapping	33 (1.9)	1.67 (1.22)	1–6	9.6%
Thermo-/cryotherapy	67 (3.9)	1.87 (1.56)	1–10	19.5%
Cross friction	37 (2.1)	1.65 (1.03)	1–5	10.8%
Laser	2 (0.1)	1.00 (0.00)	1	0.6%
Total	1721(100%)	–﻿	–﻿	500.3%

Note: % Use = percentage of patients with secondary complaints receiving modality ≥1 time. IFC, interferential current; IASTM, instrument-assisted soft tissue mobilisation; PNF, proprioceptive neuromuscular facilitation

### Crosstabulations

Crosstabulation analyses were conducted for regional examination trends across sex and age groupings for both primary and secondary complaints, as presented in Tables 6 and 7. Inferential tests could not be appropriately applied due to the inclusion of multiple response variables, of complaint regional examinations, that were not mutually exclusive.

Of the 405 males and 475 females in this study, 39% of both males (n = 158/405) and females (n = 186/475) reported secondary complaints. Of the 1252 regional examinations conducted for primary complaints, 597 were for men and 655 for females, and of the 420 regional examinations conducted for secondary complaints, 194 were for men and 226 for females.

The regional examinations conducted between sexes were relatively consistent across complaints, as seen in Table 6. However, in primary complaints, more shoulder (5.7%) and foot/ankle regionals (5%) were conducted for males than females (3.4% and 2.3%). In secondary complaints, predominant regions were lumbar (35.8%, n = 81/226) for females, and cervical for males (33.5%, n = 65/194). Regarding the age groups: 41.7% (n = 124/297) aged < 30 years; 34.7% (n = 107/308) aged 30–49 years; and 41% (n = 113/275) aged ≥ 50 years presented with secondary complaints. Again, regional examinations conducted between age groups were relatively consistent across complaints, with predominantly lumbar and cervical regional examinations being reported, shown in Table 7.

**Table 6:** Distribution of the primary (n = 1252) and secondary (n = 420) regional examinations by sex n (%)

Complaint region	Sex		
Primary complaint regions	Male (n = 405)	Female (n = 475)	Total regionals
Cervical	190 (31.8)	239 (36.5)	429
Lumbar	270 (45.2)	304 (46.4)	574
Shoulder	34 (5.7)	22 (3.4)	56
Hip	22 (3.7)	26 (4.0)	48
Elbow	5 (0.8)	2 (0.3)	7
Knee	40 (6.7)	40 (6.1)	80
Hand and Wrist	6 (1.0)	7 (1.1)	13
Foot and Ankle	30 (5.0)	15 (2.3)	45
Total regionals	597	655	1252
Secondary complaint regions	Male (n = 158)	Female (n = 186)	Total regionals
Cervical	65 (33.5)	70 (31.0)	135
Lumbar	48 (24.7)	81 (35.8)	129
Shoulder	23 (11.9)	15 (6.6)	38
Hip	11 (5.7)	12 (5.3)	23
Elbow	3 (1.5)	1 (0.4)	4
Knee	23 (11.9)	24 (10.6)	47
Hand and Wrist	6 (3.1)	7 (3.1)	13
Foot and Ankle	15 (7.7)	16 (7.1)	31
Total regionals	194	226	420

Note: Values presented as n (%). Column percentages. Inferential tests not applied due to multiple-response data

**Table 7:** Distribution of primary (n = 1252) and secondary (n = 420) regional examinations by age groups n (%)

Complaint region	Age groups			
Primary complaint regions	< 30 years (n = 297)	30–49 years (n = 308)	≥ 50 years (n = 275)	Total regionals
Cervical	168 (41.2)	145 (32.4)	116 (29.3)	429
Lumbar	166 (40.7)	208 (46.4)	200 (50.5)	574
Shoulder	19 (4.7)	21 (4.7)	16 (4.0)	56
Hip	7 (1.7)	19 (4.2)	22 (5.6)	48
Elbow	2 (0.5)	2 (0.4)	3 (0.8)	7
Knee	28 (6.9)	30 (6.7)	22 (5.6)	80
Hand and Wrist	3 (0.7)	4 (0.9)	6 (1.5)	13
Foot and Ankle	15 (3.7)	19 (4.2)	11 (2.8)	45
Total regionals	408	448	396	1252
Secondary complaint regions	< 30 years (n = 124)	30–49 years (n = 107)	≥ 50 years (n = 113)	Total regionals
Cervical	52 (35.1)	45 (33.6)	38 (27.5)	135
Lumbar	53 (35.8)	36 (26.9)	40 (29.0)	129
Shoulder	15 (10.1)	13 (9.7)	10 (7.2)	38
Hip	5 (3.4)	5 (3.7)	13 (9.4)	23
Elbow	0 (NA)	2 (1.5)	2 (1.4)	4
Knee	14 (9.5)	14 (10.4)	19 (13.8)	47
Hand and Wrist	4 (2.7)	4 (3.0)	5 (3.6)	13
Foot and ankle	5 (3.4)	15 (11.2)	11 (8.0)	31
Total regionals	148	134	138	420

Note: Values presented as n (%). Column percentages. Age groups: <30, 30–49, ≥50 years. Inferential tests not applied due to multiple-response data

### Normality and correlation

Exploring the relationships between patient age and the number of treatment sessions received for the primary and secondary complaints, Kolmogorov-Smirnov testing indicated these variables demonstrated non-normal distributions. Consequently, Spearman’s rank-order correlations were utilised for analyses. Findings indicated a weak yet significant positive correlation (ρ = 0.151, *p* = 0.005, n = 344) between age and the number of treatment sessions for secondary complaints.

## Discussion

This study provides an updated profile of patients attending a South African educational institution-based chiropractic clinic, demonstrating a stable, adult-predominant, spine-centred case mix, brief treatment episodes, and consistently multimodal, manual therapy-focused management. Compared with earlier UJCC data and international training clinic reports, the present findings suggest continuity in core patient characteristics alongside notable shifts in multiregional presentations and expanded modality use, with important implications for student clinical training.

### Demographic profile

The demographic of patients in 2023 closely mirrors earlier UJCC and other chiropractic teaching clinic studies, with a slight female predominance and a mean age in the early forties [4, 5, 9, 10, 13, 14]. The previous UJCC study of 865 new patients in 2016 reported a mean patient age of 40.9 (± 17.87) years, with the largest cohort aged 20–24 years (14.4%; n = 133).

The present findings demonstrate notable stability and suggest the clinic continues to attract a broadly similar population, despite post-pandemic shifts in healthcare utilisation [15, 16]. The sizeable contingent of younger adults likely reflects the co-located university student body, whereas the meaningful representation of older adults aligns with the greater prevalence of musculoskeletal conditions experienced by adults [17, 18]. By contrast, paediatric (0–17 years) and geriatric (≥ 65 years) encounters remained relatively infrequent, as in previous training-clinic studies [4, 5, 9, 12]. However, students may receive direct exposure to these age groups outside the clinic through targeted outreach initiatives designed to supplement chiropractic students’ exposure to less frequently encountered age groups [19–21]. Table 8 summarises the demographic findings of similar chiropractic training clinic studies.

**Table 8:** Comparison of previous studies related to demographic patient profiles within chiropractic training clinics

Author (Year)	Study location	Sample period	Sample size (n)	Mean age in years (SD)	Female (%)	Male (%)
Current study	South Africa	Jan–Jun 2023	880	40.9 (17.51)	54%	46%
Hoffman [13]	South Africa	Jan–Jul 2016	817	41.11 (17.92)	54.7%	45.3%
Ismail et al. [4]	South Africa	Jan–Jul 2016	865	40.9 (17.9)	NR	NR
Kaeser et al. [14]	USA	Oct 2013	224	37.3	47.3%	52.7%
Kioh et al. [5]	Malaysia	Aug 2018–Jul 2019	1451	34.3 (16.6)	51.1%	48.9%
Lishchyna and Mior [9]	Canada	Jul 2006–Jul 2008	580	43 (18)	57.7%	42.3%
Martinez et al. [10]	Mexico	2005–2007	500	43.4 (15.9)	61.2%	38.8%
Ricci [11]	Australia	Dec 2015–Nov 2016	325	34.3 (15.3)	56.5%	43.5%
Stevens et al. [12]	USA	Jan 2008–﻿Dec 2009	343	24.3	48.0%	52.0%

Note: n, number; SD, standard deviation; NR, not recorded

### Complaint stages, regions, and multiregional examinations

Across complaint stages, lumbar and cervical regions dominated clinical presentations, consistent with previous studies that similarly observed primarily spine-related complaints with less prevalent extremity cases becoming more frequent in subsequent complaint presentations [4, 11], supporting extensive evidence that spine-related presentations remain the predominant reason patients seek chiropractic care [22, 23]. However, in contrast to the earlier UJCC study, which recorded relatively few secondary complaints [4], the present study identified substantially more reported secondary complaints. This likely reflects a combination of increased multiregional symptom reporting, greater awareness and utilisation of chiropractic services, and more systematic documentation of subsequent presentations.

On average, each primary and secondary complaint elicited more than one regional examination, indicating routine multiregional assessment, with spinal and other associated regions often assessed in combination. This pattern is congruent with regional interdependence models that emphasise biomechanical and neurophysiological linkages between spinal and extremity regions [24, 25]. At this training clinic, interns are routinely encouraged during clinical supervision to assess beyond the symptomatic region and to integrate regional interdependence principles into clinical reasoning and management planning. The presence of multiregional examinations suggests a clinical training approach that encourages students to actively explore possible contributory regions rather than managing isolated local symptoms. Regarding education, this multiregional approach supports the development of broader diagnostic reasoning and manual skills across both spinal and extremity case presentations during clinical training. Table 9 shows comparisons of studies that recorded regions of complaint across complaint stages.

**Table 9:** Comparison of previous studies related to complaint regions of primary, secondary, and tertiary cases in chiropractic training clinics

Complaint regions	Study				
	Current study	Ismail et al. [4]	Martinez et al. [10]	Ricci [11]	Stevens et al. [12]
Primary (n)	880	865	500	325	343
Cervical	34.3%	28%	16.4%	23.4%	23%
Lumbar	45.8%	42.2%	29.2%	39.4%	51%
Upper extremity	6.1%	8.2%	(combined 28%)	8.9%	3%
Lower extremity	13.8%	16.4%		10.2%	3%
Secondary (n)	344	70	281	188	114
Cervical	32.1%	25.7%	5.3%	24%	31.6%
Lumbar	30.7%	30%	27.4%	24%	32.5%
Upper extremity	13.1%	7.1%	(combined 17.1%)	10.5%	7%
Lower extremity	24.1%	32.9%		25%	2.6%
Tertiary (n)	130	NR	76	47	21
Cervical	22.9%	–	2.6%	10.6%	14.3%
Lumbar	36%	–	17.1%	17%	19%
Upper extremity	10.4%	–	(combined 25%)	23.9%	9.5%
Lower extremity	30.7%	–		29.8%	14%

Note: n, number; NR, not recorded. Thoracic, other, and systemic categories from previous studies excluded for comparability

### Treatment episode length and course of care

Treatment episodes for both primary and secondary complaints were typically brief, with only small cohorts receiving ten or more treatment sessions per complaint. Similar findings have been consistently reported in chiropractic training clinic studies [4, 9, 12]. The relationship between treatment dose/frequency and clinical benefit is complex and condition dependent: although greater session numbers may be associated with improved outcomes [26], other reviews suggest that treatment frequency does not necessarily determine clinical efficiency [27]. In an educational clinic setting, brief episodes may also be influenced by rapid symptom improvement in acute cases, patient scheduling constraints, or a clinical focus on initial assessment and self-management planning.

Multiple factors may contribute to the brief treatment episodes observed. Educational clinics typically manage a mix of acute, subacute, and chronic presentations [5, 11, 12], in which rapid improvement may be common, depending on patient age and condition severity or complexity. In a student-run clinic, appointment availability and limited operating periods may also constrain follow-up attendance; additional considerations include patient satisfaction, time and financial constraints, clinic location and logistics, perceived clinical efficiency, and referral to another healthcare professional [28, 29]. The brevity of treatment episodes observed is not necessarily associated with undertreatment and is likely a result of a combination of factors. The small subgroup of patients with extended courses of care likely represents those with chronic, recurrent, or more complex conditions requiring ongoing conservative management, particularly among older adults [18, 30].

### Multimodal, manual-therapy-focused management and evolving modality use

Treatment at the UJCC remained firmly manual-therapy-centred, with spinal and extremity manipulation/mobilisation forming the backbone of care and being combined, in most cases, with soft tissue techniques and selected electrophysical modalities. Accordingly, the recorded treatment profile reflects a predominantly manual-therapy-centred, hands-on approach to patient management profiles that aligns with the ethos of chiropractic care [22, 31]. Compared to the earlier UJCC study [4], the current study presents an expanded record of modality implementation over multiple complaint presentations and although the recorded modality profile appears predominantly passive, this likely reflects documentation structure rather than the true absence of active care.

Multimodality treatment, on average, incorporates four to five distinct modalities over the course of a patient’s complaint, suggesting that students utilise a broad range of treatment interventions. The slight increase in the use of modality in secondary complaints possibly indicates concurrent treatment of previous complaints or the implementation of additional modalities in subsequent treatments. The most utilised modalities across complaint stages (manipulation/mobilisation; massage; myofascial dry needling) were consistent with previous training clinic studies and global scoping reviews of the chiropractic profession [4, 22]. The increased mean use of myofascial dry needling in secondary complaint cases suggests that students may increase their use of dry needling for persisting symptoms in subsequent complaints. Shockwave therapy, previously absent in earlier UJCC studies, now appears as a notable auxiliary modality that similarly increased in mean use in subsequent complaints, which may reflect integration into the chiropractic curricula, acquisition of new clinic technology, and evolving practice patterns in the management of tendinopathies and chronic soft-tissue disorders with a growing evidence base supporting its effectiveness [32].

Conversely, laser therapy remained rarely utilised, echoing local and international reports of low uptake despite its availability [4, 33, 34]. Collectively, these findings reflect the diverse clinical management strategies within UJCC and portray contemporary, conservative patient care that utilises multimodal, manual-therapy-focused approaches within the South African scope-of-practice expectations for chiropractic (Allied Health Professions Act 63 of 1982, 2001) at an educational institution-based chiropractic clinic [35].

### Associations between demographics and clinical presentation

Although the study was primarily descriptive, limited inferential analyses were conducted to explore potential associations between demographic variables and treatment-session frequency. These analyses were exploratory in nature and intended to identify patterns warranting further investigation rather than to establish causality. Sex- and age-related patterns of examined regions were largely even across complaint stages, indicating minimal variation between demographic groups and supporting the notion that students receive exposure to diverse patient populations across complaint stages. Inferential tests could not be appropriately applied due to the presence of multiregional examinations. Minor differences, such as slightly more shoulder and foot/ankle examinations among males, and relatively more lumbar region involvement among females, are consistent with known sex-specific patterns of musculoskeletal pain [36].

Among younger adults, more cervical examinations were reported, whereas lumbar examinations predominated in middle- and older-age groups across complaint stages. This aligns with likely high-activity and sport-related complaints in younger populations and age-related degenerative changes contributing to older adults’ regional complaints [17, 37]. The weak but statistically significant positive correlation between age and the number of treatment sessions for secondary complaints (ρ = 0.151, *p* = 0.005) suggests slightly longer care trajectories among older patients. This may reflect increased chronicity, degenerative changes, multimorbidity, slower recovery rates, or perceived need for ongoing conservative management once rapport has been established and care remains affordable [18, 30]. However, given the small effect size and absence of chronicity or severity data, this association should be interpreted cautiously.

These broadly even yet subtly differentiated patterns indicate that students are receiving exposure to a demographically diverse spectrum of primarily spinal and extremity-related case presentations across the adult lifespan, supporting generalisable clinical competence, while indicating that students may benefit from supplemented exposure or targeted simulations of less frequent age groups, such as paediatrics and geriatric populations.

### Educational implications

As a teaching clinic, the UJCC case mix and management patterns provide a practical indicator of the clinical exposure available to interns and the competencies most frequently reinforced in routine training. The predominance of spinal and extremity presentations, frequent multi-regional examinations, and repeated follow-up encounters suggest regular opportunities to develop core skills in history taking, clinical reasoning, region-specific examination, manual therapy delivery, and longitudinal patient management. From a programme-evaluation perspective, these data can assist in benchmarking student exposure to complaint regions across complaint stages and informing curriculum planning, supervision priorities, and clinic-based competency development. In addition, the study highlights the importance of consistent, standardised documentation, particularly for active care and self-management elements, to support both quality assurance and monitoring of patient care within a training environment.

### Strengths and limitations

This study draws on a large, consecutive sample (n = 880) with clear inclusion/exclusion criteria, thereby enhancing precision and reducing sampling bias. A piloted, structured data-collection form with predefined codes supported consistent extraction, while ethics approvals, gatekeeper permissions and de-identification ensured robust governance. The study’s scope is novel for this setting, extending beyond earlier studies: primary, secondary, and tertiary complaint stages were captured, with regional examinations and expanded modality use reported by stage. Treatment-session attendance was triangulated across signature sheets and SOAP/regional forms, and follow-up extended beyond the index visit window, allowing descriptions of care trajectories. Statistical choices were appropriately cautious, with normality checks and recognition of multiple-response structures that precluded the use of specific inferential tests.

This sample represented only the new patients who presented early in 2023 and not existing patients receiving continued care. As a retrospective, single-site chart review, findings are vulnerable to incomplete documentation and may not generalise beyond a South African university clinic. The restriction to a six-month period may not account for potential seasonal variation in presentations or clinic utilisation; however, the consecutive inclusion of all new patients and alignment with previous study timeframes enhances comparability and likely provides a representative snapshot of routine academic clinic activity.

The UJCC utilises a paper-based filing system, and as a result 12 files could not be retrieved during data collection and were excluded. Files may have been in active clinical use, awaiting administrative processing before being re-filed, or misfiled, making them unlocatable. These retrieval constraints represent a potential source of selection bias, as unavailable records could not be screened for eligibility. The reliance on paper-based handwritten records stored within physical patient files introduced potential documentation variability; however, no files were excluded due to illegibility. Limited covariates were available or inconsistently documented, including occupation, comorbidities, diagnoses, “at home” recommendations, therapeutic exercises, treatment outcomes, referrals, and overlapping treatments for subsequent complaints. Although the recorded modality profile appears predominantly passive, this likely reflects documentation structure rather than the true absence of active care. Additional demographic variables, such as race, employment status, income, and education, are never recorded in patient files/forms and cannot be collected, thereby constraining exploratory depth.

Methodologically, the multiple-response nature of multiregional examinations restricted the use of conventional inferential tests for some comparisons. Although the total number of treatment sessions was accrued through later dates for complaint stages, and overall, specifications regarding complaint chronicity, treatment frequency intervals or duration were not recorded. Tertiary complaints were captured at a regional level only, and detailed modality/frequency data beyond this stage were not collected. The presence of each modality per session was captured and aggregated across complaint stages to explore modality usage patterns, rather than tallying multiple applications within individual treatment sessions, which precluded regional utilisation and precise frequency/dose analysis. Finally, a single extractor was used without independent dual-coding, which limits evidence of inter-rater reliability despite internal re-checks.

## Conclusion

The demographic and clinical characteristics gathered from this study show similarities to prior local and international studies of educational institution-based chiropractic clinics. This latest and expanded post-pandemic descriptive information on 880 new patients presenting in 2023 reveals a stable, adult-predominant case mix, with presenting, secondary, and tertiary complaints that commonly involved spine-related and rarely elbow regional examinations across all stages. Treatment courses were concise, and care was consistently multimodal and manual-therapy-focused, with selective use of adjunctive soft-tissue and electrophysical modalities; laser was nearly never utilised. Age was weakly associated with longer secondary-care, while sex- and age-related differences in the examined regions were small. Collectively, these findings portray contemporary, conservative chiropractic practices within scope and provide a high-resolution baseline for curriculum review, resource allocation, improvement planning, and longitudinal comparisons with other educational clinics or private practices. Future work should focus on narrowing the scope, linking the modality “dose” and visit frequency/duration to patient-centred outcomes, enriching covariates (such as race, occupation, and chronicity of complaint), and leveraging electronic systems to enable prospective, multi-site analyses that can inform both education and service delivery.

## Data Availability

The data that support the findings of this study are available on reasonable request from the corresponding author.

## References

[1] Ewnte B, Yigzaw T. Early clinical exposure in medical education: the experience from Debre Tabor University. BMC Med Educ. 2023;23(1):252. 37069522 10.1186/s12909-023-04221-4 PMC10111732

[2] Puhl AA, Reinhart CJ, Injeyan HS, Tibbles A. Description of the case mix experienced by chiropractic students during a clinical internship. J Chiropr Educ. 2017;31(2):132–9. 28657811 10.7899/JCE-16-00017 PMC5656149

[3] Haworth NG, Horstmanshof L, Moore KM. Chiropractic and osteopathic students’ perceptions of readiness for transition to practice: The educational value of university clinic vs community and private clinics. J Chiropr Educ. 2021;35(1):38–49. 32543901 10.7899/JCE-19-13 PMC7958668

[4] Ismail F, Booysen N, Yelverton C, Peterson C. Characteristics of chiropractic patients treated at the University of Johannesburg chiropractic student clinic and relevance to the educational process. J Chiropr Educ. 2021;35(2):215–21. 33316062 10.7899/JCE-19-29 PMC8528433

[5] Kioh SH, Pooke TG, Chong SF. Demographics and clinical profiles of patients visiting a chiropractic teaching clinic in Malaysia. J Chiropr Med. 2021;20(3):115–20. 35463846 10.1016/j.jcm.2021.12.001 PMC9023127

[6] Ford TW. Chiropractic and public health: a study on the perceptions and attitudes of chiropractors on health promotion and disease prevention in South Africa. Durban, South Africa: Durban University of Technology, 2014.

[7] Myburgh C, Mouton J. Developmental issues in chiropractic: a South African practitioner and patient perspective. J Manip Physiol. Ther. 2007;30(3):206–14. 17416275 10.1016/j.jmpt.2007.01.004

[8] Chiropractic Association of South Africa. Available from https://chiropractic.co.za/student-information/

[9] Lishchyna N, Mior S. Demographic and clinical characteristics of new patients presenting to a community teaching Clinic. J Chiropr Educ. 2012;26(2):161–8. 23362363 10.7899/JCE-12-002 PMC3557651

[10] Martinez DA, Rupert RL, Ndetan HT. A demographic and epidemiological study of a Mexican chiropractic college public clinic. Chiropr Man Ther. 2009;17(1):4. 10.1186/1746-1340-17-4 PMC2667450 19298656

[11] Ricci M. A comparison of the demographic and clinical characteristics of a chiropractic teaching clinic and private practice. Murdoch University; 2019.

[12] Stevens G, Campeanu M, Sorrento AT, Ryu J, Burke J. Retrospective demographic analysis of patients seeking care at a Free University chiropractic clinic. J Chiropr Med. 2016;15(1):19–26. 27069428 10.1016/j.jcm.2016.02.001 PMC4812027

[13] Hoffman C. Clinical audit of new patients consulting at the University of Johannesburg Chiropractic Clinic. Johannesburg: University of Johannesburg; 2018.

[14] Kaeser MA, Hawk C, Anderson M. Patient characteristics upon initial presentation to chiropractic teaching clinics: a descriptive study conducted at one university. J Chiropr Educ. 2014;28(2):146–51. 25162982 10.7899/JCE-14-6 PMC4211587

[15] Alegre JC, Sharma S, Cleghorn F, Avila C. Strengthening primary health care in low- and middle-income countries: furthering structural changes in the post-pandemic era. Front Public Health. 2024;11:1270510. 38419816 10.3389/fpubh.2023.1270510 PMC10899890

[16] Moynihan R, Sanders S, Michaleff ZA, Scott AM, Clark J, To EJ, Jones M, Kitchener E, Fox M, Johansson M, et al. Impact of COVID-19 pandemic on utilisation of healthcare services: a systematic review. BMJ Open. 2021;11(3):e045343. 33727273 10.1136/bmjopen-2020-045343 PMC7969768

[17] Blyth FM, Noguchi N. Chronic musculoskeletal pain and its impact on older people. Best Pract Res Clin Rheumatol. 2017;31(2):160–8. 29224694 10.1016/j.berh.2017.10.004

[18] Kamerman PR, Bradshaw D, Laubscher R, Pillay-van Wyk V, Gray GE, Mitchell D, Chetty S. Almost 1 in 5 South African adults have chronic pain: a prevalence study conducted in a large nationally representative sample. Pain. 2020;161(7):1629–35. 32102020 10.1097/j.pain.0000000000001844

[19] Frederick TM, Varatharajullu D, Sibiya MN. Perceptions of new graduate chiropractors in their management of paediatric patients in the eThekwini municipality. Global J Health Sci. 2020;12(3):32.

[20] Naidoo K. The clinical experience of registered master’s chiropractic students in the management of elderly patients during their practicum. Durban: Durban University of Technology; 2021.

[21] Todd AJ, Carroll MT, Russell DG, Mitchell EKL. A prospective survey of chiropractic student experiences with pediatric care and variability of case mix while on clinical placement in Rarotonga. J Chiropr Educ. 2017;31(1):14–9. 27967212 10.7899/JCE-16-4 PMC5345780

[22] Beliveau PJH, Wong JJ, Sutton DA, Simon NB, Bussières AE, Mior SA, French SD. The chiropractic profession: a scoping review of utilization rates, reasons for seeking care, patient profiles, and care provided. Chiropr Man Ther. 2017;25(1):35. 10.1186/s12998-017-0165-8 PMC5698931 29201346

[23] Gevers-Montoro C, Provencher B, Descarreaux M, Ortega De Mues A, Piché M. Clinical effectiveness and efficacy of chiropractic spinal manipulation for spine pain. Front Pain Res. 2021;2:765921. 10.3389/fpain.2021.765921 PMC8915715 35295422

[24] McDevitt A, Young J, Mintken P, Cleland J. Regional interdependence and manual therapy directed at the thoracic spine. J Man Manip Therapy. 2015;23(3):139–46. 10.1179/2042618615Y.0000000005 PMC4534849 26309384

[25] Sueki DG, Cleland JA, Wainner RS. A regional interdependence model of musculoskeletal dysfunction: research, mechanisms, and clinical implications. J Man Manip Therapy. 2013;21(2):90–102. 10.1179/2042618612Y.0000000027 PMC3649356 24421619

[26] Haas M, Vavrek D, Peterson D, Polissar N, Neradilek MB. Dose-response and efficacy of spinal manipulation for care of chronic low back pain: a randomized controlled trial. Spine J. 2014;14(7):1106–16. 24139233 10.1016/j.spinee.2013.07.468 PMC3989479

[27] Pasquier M, Daneau C, Marchand AA, Lardon A, Descarreaux M (2019) Spinal manipulation frequency and dosage effects on clinical and physiological outcomes a scoping review. Chiropr Man Ther, 27(1), p. 23. 10.1186/s12998-019-0244-0 PMC6530068 31139346

[28] Boylan P. Factors that contribute to the perceived treatment effect of spinal manipulative therapy in a chiropractic teaching clinic: a qualitative study. Chiropr Man Ther. 2024;32(1):41. 10.1186/s12998-024-00554-z PMC11658066 39695654

[29] Mohammed Selim S, McPhail SM, Carter HE, Malatzky C, Kularatna S, Naicker S. We’re here to help them if they want to come: a qualitative exploration of hospital staff perceptions and experiences with outpatient non-attendance. PLoS ONE. 2025;20(6):e0311059. 40489520 10.1371/journal.pone.0311059 PMC12148132

[30] Cohen SP, Vase L, Hooten WM. Chronic pain: an update on burden, best practices, and new advances. Lancet. 2021;397(10289):2082–97. 34062143 10.1016/S0140-6736(21)00393-7

[31] Glucina TT, Krägeloh CU, Spencer K, Holt K. Defining chiropractic professional identity: a concept analysis. J Bodyw Mov Ther. 2023;35:75–83. 37330807 10.1016/j.jbmt.2023.04.047

[32] Crevenna R, Mickel M, Schuhfried O, Gesslbauer C, Zdravkovic A, Keilani M. Focused extracorporeal shockwave therapy in physical medicine and rehabilitation. Curr Phys Med Rehabilitation Rep. 2021;9(1):1–10.

[33] Csiernik B, Smith A, Plener J, Tibbles A, Young JJ. Intervention usage for the management of low back pain in a chiropractic teaching clinic. Chiropr Man Ther. 2022;30(1):3. 10.1186/s12998-022-00412-w PMC8743057 35000607

[34] Morgan E. The prevalence and types of non-manipulative therapies used by chiropractors in South Africa. Johannesburg: University of Johannesburg; 2017.

[35] Africa AHPCS. Allied Health Professions Act, 1982 (Act No. 63 of 1982). In: Allied Health Professions Council of South Africa; 1982.

[36] Wáng YXJ, Wáng J-Q, Káplár Z. Increased low back pain prevalence in females than in males after menopause age: evidences based on synthetic literature review. Quant Imaging Med Surg. 2016;6(2):199–206. 27190772 10.21037/qims.2016.04.06 PMC4858456

[37] Tan A, Strauss VY, Protheroe J, Dunn KM (2018) Epidemiology of paediatric presentations with musculoskeletal problems in primary care. BMC Musculoskelet Disord, 19(1), 40. 29409492 10.1186/s12891-018-1952-7 PMC5801684

